# Prognostic Value of [18F]-FDG PET/CT Radiomics Combined with Sarcopenia Status among Patients with Advanced Gastroesophageal Cancer

**DOI:** 10.3390/cancers14215314

**Published:** 2022-10-28

**Authors:** Ricarda Hinzpeter, Seyed Ali Mirshahvalad, Roshini Kulanthaivelu, Claudia Ortega, Ur Metser, Zhihui A. Liu, Elena Elimova, Rebecca K. S. Wong, Jonathan Yeung, Raymond Woo-Jun Jang, Patrick Veit-Haibach

**Affiliations:** 1 Joint Department of Medical Imaging, Toronto General Hospital, University Health Network, University of Toronto, Toronto, ON M5G 2C4, Canada; 2 Department of Biostatistics, Princess Margaret Cancer Centre, University Health Network, University of Toronto, Toronto, ON M5G 2C4, Canada; 3 Department of Medical Oncology, Princess Margaret Cancer Centre, University Health Network, University of Toronto, Toronto, ON M5G 2C4, Canada; 4 Department of Radiation Oncology, Princess Margaret Cancer Centre, University Health Network, University of Toronto, Toronto, ON M5G 2C4, Canada; 5 Division of Thoracic Surgery, Department of Surgery, Toronto General Hospital, University Health Network, University of Toronto, Toronto, ON M5G 2C4, Canada

**Keywords:** ^18^[18F]-FDG PET/CT, radiomics, sarcopenia, gastroesophageal cancer

## Abstract

**Simple Summary:**

Increasing knowledge of outcome prognostication, derived from various clinical and imaging-derived parameters, has the potential for pretreatment risk stratification, allowing clinicians to deliver more patient specific treatment tailored to individual risk. Our study indicates that a prognostic model, combining clinical parameter, PET and CT radiomics with sarcopenic status, derived from standard of care clinical 18F-FDG-PET/CT improves outcome prognostication among patients with advanced, metastatic esophageal and gastroesophageal cancer.

**Abstract:**

We investigated, whether ^18^[18F]-FDG PET/CT-derived radiomics combined with sarcopenia measurements improves survival prognostication among patients with advanced, metastatic gastroesophageal cancer. In our study, 128 consecutive patients with advanced, metastatic esophageal and gastroesophageal cancer (n = 128; 26 females; 102 males; mean age 63.5 ± 11.7 years; age range: 29–91 years) undergoing ^18^[18F]-FDG PET/CT for staging between November 2008 and December 2019 were included. Segmentation of the primary tumor and radiomics analysis derived from PET and CT images was performed semi-automatically with a commonly used open-source software platform (LIFEX, Version 6.30, lifexsoft.org). Patients’ nutritional status was determined by measuring the skeletal muscle index (SMI) at the level of L3 on the CT component. Univariable and multivariable analyses were performed to establish a survival prediction model including radiomics, clinical data, and SMI score. Univariable Cox proportional hazards model revealed ECOG (<0.001) and bone metastasis (*p* = 0.028) to be significant clinical parameters for overall survival (OS) and progression free survival (PFS). Age (*p* = 0.017) was an additional prognostic factor for OS. Multivariable analysis showed improved prognostication for overall and progression free survival when adding sarcopenic status, PET and CT radiomics to the model with clinical parameters only. PET and CT radiomics derived from hybrid ^18^[18F]-FDG PET/CT combined with sarcopenia measurements and clinical parameters may improve survival prediction among patients with advanced, metastatic gastroesophageal cancer.

## 1. Introduction

Gastroesophageal cancer ranks seventh most common malignancy worldwide, with approximately 600,000 new cases annually, accounting for the sixth most common cause of cancer-associated death [1]. Despite the ongoing development of therapeutic strategies, the current prognosis remains poor with a 5-year survival rate ranging between 5–46%. For patients with early stage, resectable gastroesophageal cancer, surgical treatment is considered a curative approach, often following neoadjuvant chemotherapy or chemoradiotherapy [2]. However, approximately 50% of patients present with locally advanced disease, which often render those patients palliative candidates, who are then typically treated with chemotherapy [3,4]. Recent studies also investigated the role of immunotherapeutic agents in gastroesophageal cancer, showing promising results regarding efficacy, tolerable toxicity and survival rate in adequately selected patients [5,6].

Fluorine-18-Fluorodeoxyglucose positron emission tomography/computed tomography ([18F]-FDG PET/CT) plays an important role in staging and treatment response assessment in various malignancies, including gastroesophageal cancer, providing significant diagnostic and prognostic value amongst these patients [7,8,9]. [18F]-FDG PET/CT provides various different standard metabolic parameters, including standardized uptake value (SUV), metabolic tumor volume (MTV) or total lesion glycolysis (TLG). However, these values do not show the underlying spatial distribution of tracer activity within the primary, and thus tumor heterogeneity is not accounted for in imaging analysis.

In recent years, ongoing developments of machine learning techniques and the huge growth of computational power have driven the field of radiomics [10]. The principle of radiomics includes the extraction of high-dimensional data from various sources of medical images, possibly providing incremental information on underlying pathophysiology, aiming to support the clinical decision-making [7,11,12]. The results of several studies investigating intra-tumoral heterogeneity on [18F]-FDG PET/CT in patients with gastroesophageal cancer, indicate that textural parameters may improve prognostication of treatment response and prognosis, especially when combined with clinical data [7,13,14,15,16].

Sarcopenia describes the poor nutritional status, characterized by an involuntary loss of muscle mass, leading to increased morbidity and mortality [17]. Sarcopenia has been found to be a poor prognostic survival factor in several cancer patient populations, especially in patients with gastroesophageal cancer [18,19].

Radiomics and sarcopenia measurements have been used so far independently for risk stratification and survival prognostication. Thus, the aim of our study was to determine the prognostic value of combining radiomics parameters from [18F]-FDG PET/CT with patient’s sarcopenic status among patients with advanced, metastatic esophageal and gastroesophageal cancer.

## 2. Materials and Methods

128 consecutive patients with primary metastatic esophageal or gastroesophageal cancer, who underwent [18F]-FDG PET/CT between November 2008 and December 2019, as part of their initial staging, were included in the study. 35 patients were excluded from the study, due to missing baseline [18F]-FDG PET/CT. Overall demographic data are provided in Table 1.

This study was conducted after institutional and local ethics committee approval (REB# 19-5575). The requirement for informed consent was waived.

### 2.1. Imaging Acquisition

[18F]-FDG PET/CT acquisitions were performed on a Siemens mCT40 (Siemens Healthineers, Erlangen, Germany) with 5–9 bed potions, depending on patient’s height (2.5 min acquisition time). Patients received 300–400 Mbq (4–5 MBq/kg) of [18F]-FDG 60 min prior to image acquisition. Oral contrast media was administered for bowel opacification; no intravenous contrast media was used. Images were obtained from the skull base to the upper thighs. CT as part of PET/CT was performed using the following scan parameters: Tube voltage 120 kVp, collimation 2 mm, rotation time 0.8 s, feed/rotation 8.4 mm. PET emission scan using time of flight with scatter correction was obtained covering the identical transverse field of view. PET parameters were as follows: image size: 2.6 pixels; slice: 3.27; and 4-mm full width at half maximum (FWHM) gaussian filter type.

### 2.2. Image Analysis and Sarcopenia Measurements

Image analysis was performed manually by one radiologist with 5 years of experience in oncologic imaging, using a common, commercially available image software (Mirada XD Workstation, Mirada Medical, Ltd.; Oxford, UK). Standard metabolic parameter, including mean, max and peak standardized uptake value (SUV) normalized by lean body mass (SUL) was measured in the primary tumor for each patient. Sarcopenia measurements were calculated from the CT component of the [18F]-FDG PET/CT by the same reader. Assessment of skeletal muscle mass at the level of the third lumbar vertebra was performed with −29–150 HU thresholds, using Slice-O-Matic (TomoVision, version 5.0, Magog, QC, Canada). Skeletal muscle index (SMI) was calculated by normalizing the muscle area (cm^2^) for patient’s height in squared meters (m^2^). Sarcopenia cutoff values were used as follows [20]: SMI of 34.4 cm^2^/m^2^ in females and SMI of 45.4 cm^2^/m^2^ in males.

### 2.3. Image Segmentation and Radiomic Feature Extraction

**Figure 1:**
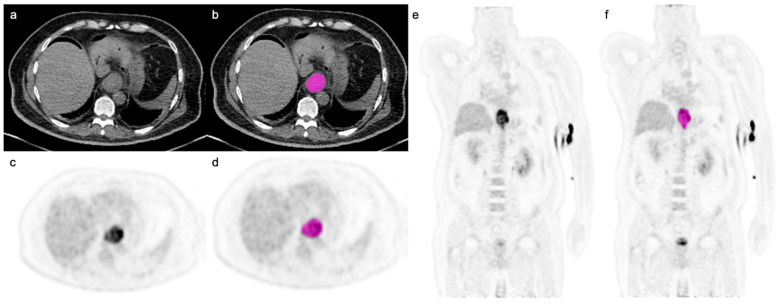
Axial CT image (**a**) and axial and coronal PET images (**c**,**e**) with segmentation (displayed in pink; (**b**,**d**,**f**)) in a 60-year-old male patient with poorly differentiated gastroesophageal adenocarcinoma (SUVmax 16.7; SUVmean 8.4). Note is made of free intraperitoneal gas in the upper abdomen due to recent insertion of a gastrostomy tube.

Images segmentation was performed using a commonly available open-source software (LIFEx, version 6.30; lifexsoft.org [21]). The primary tumor was segmented semi-automatically on the PET component of the study, using a thresholding-based approach, applying three different thresholds on the PET volumes of interest (VOI), defined as (1) Background, (2) 40% and (3) 70% of the maximal SUV of the defined lesion, as previously described [21,22]. Volumetric segmentation of the primary tumor on the CT component was carried out manually in a slice-by-slice fashion (Figure 1).

### 2.4. Statistical Analysis

Summary statistics were used to describe demographic and disease characteristics. Kaplan–Meier (KM) method was used to estimate overall survival (OS) and progression-free survival (PFS). Preprocessing of the data included radiomic features removal with more than 30% missing observations and removing features with little variation. All PET and CT features were standardized with a mean of 0 and a standard deviation of 1. The Univariable Cox proportional hazards model (UVA) was fitted to assess clinical variables, including demographic and disease-associated data, SUV parameters and anthropometric indices. Parameters with a *p*-value of <0.05 were included in a subsequent analysis to build a multivariable Cox model. Lastly, highly correlated features were removed with a cutoff of >0.7 to reduce pairwise correlations. To construct and choose the best performing model by Akaike information criterion (AIC), backward and forward stepwise Cox regression was conducted, with the full model consisting of the selected clinical variables and all the radiomic features from the previous step. Model performance was quantified and visualized using area under the time-dependent receiver operating characteristic (ROC) curve (AUC), calculated using leave-one-out cross-validation which served as an internal validation method. All statistical analyses were carried out in R version 4.0.2 [23]. R package *caret* [24] was used for feature preprocessing and correlation assessment, *MASS* [25] was used for stepwise regression and model selection, and time-dependent ROC curve was generated using *survivalROC* [26].

## 3. Results

128 consecutive patients with primary metastatic esophageal or gastroesophageal squamous cell carcinoma (n = 44) and adenocarcinoma (n = 84) who underwent [18F]-FDG PET/CT as part of the initial staging, were included in this study. All patients were considered palliative, treated with chemotherapy (n = 63), radiation (n = 52) or a combination of both (n = 13). 2/128 patients underwent additional salvage esophagectomy and esophago-gastrectomy, respectively. All chemotherapy regimens included a platin-based agent, mostly combined with either capecitabine or paclitaxel/docetaxel.

Median (95% confidence interval) OS and PFS for the entire cohort were 9.0 (6.9–10.7) months and 6.0 (4.7–7.0) months, respectively. There was no significant difference between patients with squamous cell carcinoma and adenocarcinoma regarding OS and PFS (*p* = 0.67 and 0.68, respectively), thus all further results are described for the overall cohort only. Baseline characteristics are displayed in Table 1.

### 3.1. Univariable Analysis

ECOG performance status (*p* < 0.001), bone metastases (*p* = 0.028) and sarcopenia (both dichotomized sarcopenia score and SMI values; *p* = 0.033 and 0.0075, respectively) were poor prognostic factors for OS and PFS. Age was an additional prognostic factor for worse OS in the overall cohort (*p* = 0.017). Standard SUV parameters from staging [18F]-FDG PET/CT did not show significant associations with poor OS and PFS (Table 2) and were not included in the multivariable analysis.

Univariable analysis with regard to the CT component of the [18F]-FDG PET/CT revealed that NGLDM Coarseness (*p* = 0.018 and 0.013, respectively) and NGLDM_Contrast (*p* = 0.009 and 0.016, respectively) were associated with a significant decrease in OS and PFS. GLZLM_ZP (*p* = 0.048) was an additional poor prognostic parameter for OS. Analysis of the PET features revealed SHAPE_Volume_ml (*p* = 0.049), GLZLM_SZLGE (*p* = 0.043) and GLZLM_LZLGE (*p* = 0.044) as being associated with poor OS. PET radiomics features showing statistical significance regarding PFS were as follows: SHAPE_Volume_mL (*p* = 0.017), SHAPE_Volume_vx (*p* = 0.049), SHAPE_Surface_mm^2^ (*p* = 0.043), GLZLM_LZE (*p* = 0.026), GLZLM_LZLGE (*p* = 0.046), 40_ SHAPE_Volume_mL (*p* = 0.038), 70_Kurtosis (*p* = 0.042) and 70_Excess Kurtosis (*p* = 0.042) (Table 2).

### 3.2. Multivariable Analysis

On multivariable analysis ECOG performance status (*p* < 0.001) and bone metastases (*p* = 0.021 and 0.005, respectively) remained statistically significant clinical prognostic factors for worse OS and PFS (Table 3). With regard to CT features, NGLDMCoarseness was the most significant feature for OS and NGLDM_Contrast was the most significant feature for PFS (both *p* = 0.01). From the PET features GLZLM_SZLGE was most statistically significant for OS (*p* = 0.002). SHAPE_Volume_vx and 70_ Kurtosis were the most statistically significant predictors for PFS (*p* = 0.04 and 0.05, respectively).

**Figure 2:**
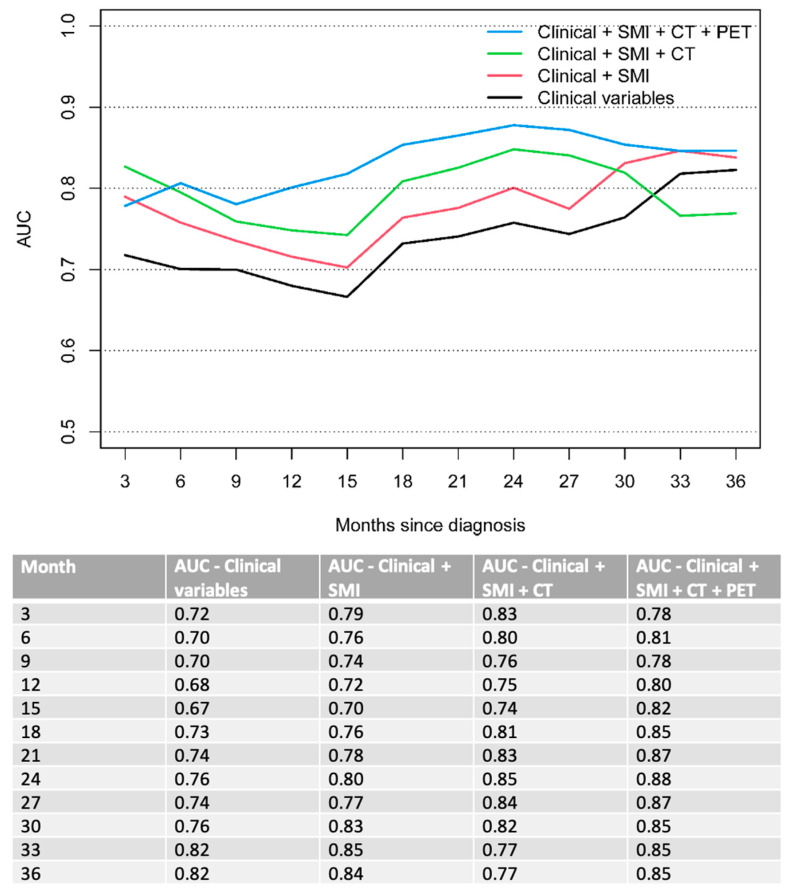
Time-dependent AUC for OS. Clinical variables include ECOG performance status and bone metastases.

Subsequently, a combined clinical model was created, stepwise adding sarcopenic status of the patient as well as independent CT and PET features, improving the accuracy of the model with each additional parameter. Overall, the combined model (clinical + SMI + CT + PET) outperformed all other models (solely clinical vs. clinical + SMI vs. clinical + SMI + CT) for the prediction of OS over a clinical course of 6 to 33 months of follow-up. OS AUC 0.7 vs. 0.76 vs. 0.8 vs. 0.81 at 6 months; 0.68 vs. 0.72 vs. 0.75 vs. 0.8 at 12 months and 0.76 vs. 0.8 vs. 0.85 vs. 0.88 at 24 months (Figure 2).

**Figure 3:**
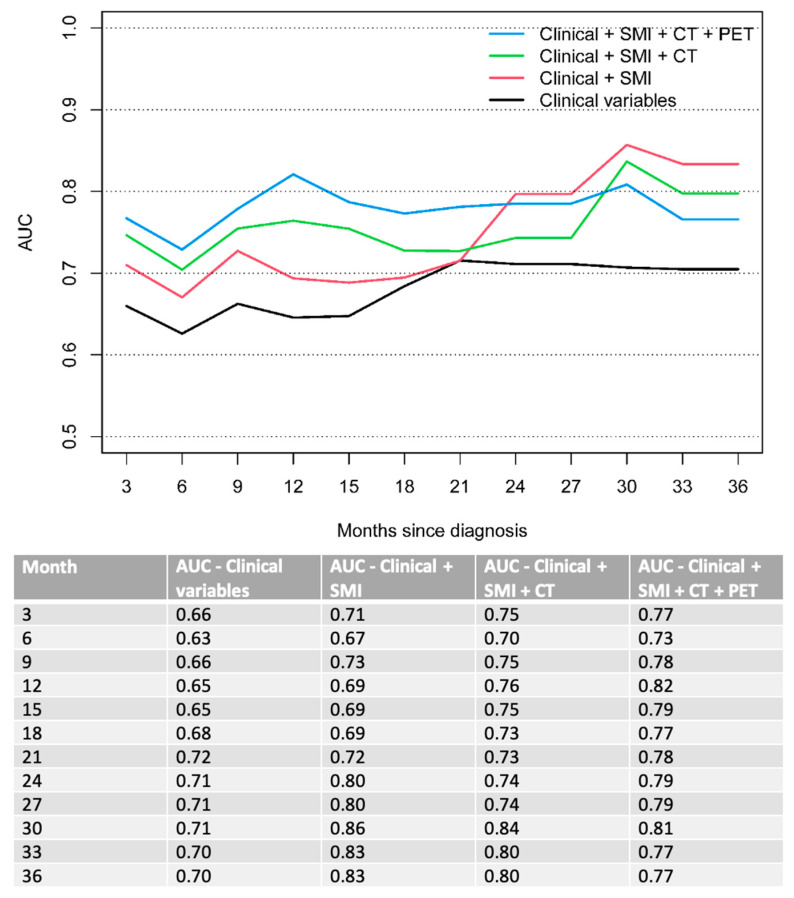
Time-dependent AUC for PFS. Clinical variables include ECOG performance status and bone metastases.

The combined model also outperformed all other models regarding PFS over a clinical course of 3 to 21 months. PFS AUC 0.63 vs. 0.67 vs. 0.7 vs. 0.73 at 6 months. 0.65 vs. 0.69 vs. 0.76 vs. 0.82 at 12 months and 0.72 vs. 0.72 vs. 0.73 vs. 0.78 at 21 months (Figure 3). At later stage disease (24–36 months), the model with combined clinical parameters and sarcopenia measurements (clinical + SMI) demonstrated the best performance for predicting PFS.

## 4. Discussion

In our study, we investigated the prognostic ability of combined [18F]-FDG PET/CT radiomics features complemented with clinical parameter and sarcopenic status among patients with advanced, metastatic esophageal and gastroesophageal cancer with regard to OS and PFS. The main finding of our study demonstrates a stepwise improvement of the survival prognostication when adding sarcopenic status, independent CT and PET features to the solely clinical model, indicating superior prognostic ability of the overall combined model for both OS and PFS.

[18F]-FDG PET/CT is an important imaging modality for staging, assessing treatment response and the detection of recurrence after treatment in patients with gastroesophageal cancer [27,28]. There is conflicting literature about the prognostic ability of quantitative metabolic measurements in terms of prognostication. While several studies suggesting standard metabolic parameters, such as SUVmean and SUVmax, can be helpful prognostic tools among patients with esophageal and gastroesophageal cancer [29,30], the results of several other studies do not support this finding, showing no improvement in outcome prediction taking into account these parameters [31,32]. More advanced volumetric parameters, including metabolic tumor volume (MTV) or total lesion glycolysis (TLG), which integrate metabolically active tumor volume with tumor FDG uptake have also been proposed as effective prognostic tools [29]. However, the FDG uptake of a primary tumor may be heterogeneously distributed, partly due to underlying pathophysiological conditions, like metabolism, hypoxia, necrosis and cellular proliferation [15,33,34]. In addition, the intra-tumoral heterogeneity can be related to tumor aggressiveness, therapy response and prognosis, and established [18F]-FDG PET/CT parameters may not fully reveal these characteristics and is not reflective of the spatial tumoral heterogeneity [33,35]. Thus, different and more advanced quantitative measures are needed to capture those underlying aspects of the tumor. In recent years, the field of radiomics, enabling the extraction of high-dimensional data from various sources of medical images, including functional imaging like PET, has shown promising results with regard to response and outcome prediction among a broad range of malignancies, including gastroesophageal cancer [36,37,38]. However, to the best of our knowledge, no study so far investigated independent CT and PET features in combination with clinical variables and sarcopenic measurements in a more holistic model for outcome prediction, among patients with advanced, metastatic esophageal and gastroesophageal cancer. Most studies so far correlated textural features with tumor stage or evaluated the ability of predicting tumor response to neoadjuvant chemoradiotherapy [38,39,40] and only very limited studies correlated textural features with survival prognostication. The reason for this may be the significantly reduced life expectancy in this patient population since those patients are treated mostly palliatively. However, with the introduction of multi-line therapy options, including immunotherapeutic agents, the prognosis in these patients may be improved over time [41]. Within the current literature, Dong et al. [36] investigated 116 patients with esophageal squamous cell carcinoma who underwent surgical resection. The authors applied an area under the cumulative SUV volume histogram (AUC-CSH) method, which might be used as a simplified, quantitative parameter of metabolic heterogeneity. The results of their study indicate that higher intra-tumoral metabolic heterogeneity may predict postoperative recurrence and survival in patients with resected primary. Similar results were found by Yip et al. [38], who evaluated a smaller cohort of 54 patients with esophageal squamous cell carcinoma and adenocarcinoma, who underwent mainly surgery after the neo-adjuvant chemoradiotherapy, showing that all textural features from [18F]-FDG PET/CT were better correlated to pathologic response and overall survival than standard metabolic parameters like SUVmax and SUVmean. For example, entropy and run-length matrix (RLM) texture features significantly discriminated patients with good and poor overall survival. This confirms the results of our study, demonstrating enhanced survival prognostication when applying radiomics features in an even larger and more homogenous patient cohort. A further difference to our study is the application of texture analysis, whereas radiomics analysis was used in our study. Foley et al. [42] showed that TLG, histogram energy and histogram kurtosis were independent predictors for worse OS in a large retrospective cohort of 403 patients with either esophageal squamous cell carcinoma or adenocarcinoma, deemed to have a potentially curable disease, following contrast-enhanced CT (CECT), however approximately 50% were considered palliative following [18F]-FDG PET/CT. When comparing to our results, certain differences and similarities can be pointed out. Our results demonstrated that coarseness and contrast from CT feature analysis and kurtosis from PET feature analysis were associated with worse OS and PFS. Similar to prior studies, including the study by Foley et al. [42], this may indicate that features which measure local intensity variations and the shape of the intensity distribution of data seem to have potential predictive value. Furthermore, we evaluated both esophageal squamous cell carcinoma and adenocarcinoma, however all patients in our cohort had advanced metastatic disease and were treated with a standard palliative therapy regimen, indicating a more homogenous study cohort. Notably, we also included both PET and CT radiomics features in our final model, whereas Foley et al. [42] applied textural analysis of PET images only.

Nakajo et al. [43] performed textural analysis on 52 patients with esophageal squamous cell carcinoma, to evaluate whether [18F]-FDG PET/CT-derived features predict response and prognosis in patients treated with neoadjuvant chemoradiotherapy prior to surgery. TLG, MTV, intensity variability and size-zone variability were independent predictors for treatment response but not for OS and PFS. Discrepancies to the results of our study may be explained by the inclusion of PET-derived radiomics features only, the smaller population and different study cohort characteristics, where we included only patients with advanced disease with distant metastases and palliative treatment intent.

Xiong et al. [44] developed a prognostic model, incorporating clinical variables in combination with textural features from pre-and mid-treatment [18F]-FDG PET/CT, demonstrating high accuracy (accuracy 93.3%, specificity 95.7, sensitivity 85.7%) for the prediction of PFS in a cohort of 30 patients with esophageal squamous cell carcinoma, treated with definite chemoradiotherapy. Our study demonstrates similar results, however we additionally/exclusively incorporated sarcopenia measurements to the final model in addition to clinical variables, independent CT and PET features, reaching stepwise improvement of the ability to predict OS and PFS, except for late stage (24–36 months) disease where the combination of only clinical variables with sarcopenic status showed the best performance with regard to PFS (AUC 0.86 (clinical + SMI) vs. 0.81 (overall combined final model)) at 30 months of follow-up. This can likely be explained by the fact that usually, patients would change to another line of therapy after progression and thus, the predictive value decreases.

The following study limitations must be acknowledged. First, there are inherent drawbacks, due to the retrospective nature of the study, the relatively small sample size and the monocentric characteristics. Second, our study lacks an external validation cohort. Third, we did not perform radiomics analysis and sarcopenia measurements on post-treatment imaging, since [18F]-FDG PET/CT is only funded for staging purposes in our current environment.

## 5. Conclusions

In conclusion, our study indicates that combined standard of care [18F]-FDG PET/CT-derived radiomics features (both CT and PET) in addition to sarcopenic status and clinical parameters has incremental value in survival prognostication among patients with metastasized esophageal and gastroesophageal cancer.

## Figures and Tables

**Table 1:** Overall demographic data.

Characteristics	n = 128
Age (mean ± SD; range)	63.5 ± 11.7 (29–91)
Sex	
Females	26 (20%)
Male	102 (80%)
BMI (kg/m^2^) (mean ± SD)	24.4 ± 4.9
Race	
Asian	11 (9%)
Non-Asian	117 (91%)
Siewert Class	
AEG 1: 35–39 cm	27 (21%)
AEG 2: 39–42 cm	27 (21%)
AEG 3: 42–45 cm	15 (12%)
Esophagus: <35 cm	59 (46%)
ECOG	
0	28 (22%)
1	73 (57%)
≥2	27 (21%)
Tumor Grade	
G1-2	47 (37%)
G3	51 (40%)
GX	30 (23%)
T stage	
T0-3	37 (29%)
T4	8 (6%)
TX	83 (65%)
N stage	
N0	6 (5%)
N1	113 (88%)
N2	4 (3%)
NX	5 (4%)
M stage	128 (100%)
Distant Lymph Nodes	73 (57%)
Lung/Pleura	24 (19%)
Liver	43 (34%)
Peritoneum	16 (12%)
Bone	29 (23%)
Brain	2 (2%)
Sarcopenia	60 (47%; 82% males, 18% females)

**Table 2:** Univariable Cox regression analysis for OS and PFS in the overall cohort.

Covariate	OS		PFS	
	HR (95%CI)	*p*-value	HR (95%CI)	*p*-value
Age	1.02 (1.00,1.04)	**0.017**	1.01 (1.00,1.03)	0.14
Sex (male)	0.90 (0.57,1.43)	0.65	1.00 (0.64,1.57)	0.99
Race (non-asian)	0.69 (0.36,1.34)	0.28	0.53 (0.27,1.02)	0.058
ECOG		**<0.001**		**<0.001**
0–1	Reference		Reference	
2–3	3.13 (1.96,4.98)		2.30 (1.46,3.62)	
T stage		0.47		0.27
T0-3	Reference		Reference	
T4	1.05 (0.44,2.55)		0.70 (0.29,1.68)	0.42
TX	1.30 (0.84,2.02)		1.25 (0.83,1.90)	0.28
Tumor Histology		0.68		0.69
Adenocarcinoma	Reference		Reference	
Squamous cell carcinoma	0.92 (0.61,1.37)		1.08 (0.74,1.59)	
Tumor Grade		0.77		0.86
G1-2	Reference		Reference	
G3	0.92 (0.60,1.41)		0.91 (0.60,1.37)	0.64
GX	1.11 (0.67,1.85)		1.02 (0.63,1.66)	0.93
M		0.44		0.45
M1	Reference		Reference	
M1a	0.70 (0.27,1.79)		0.69 (0.29,1.65)	0.4
M1b	1.18 (0.77,1.79)		1.14 (0.76,1.70)	0.53
Distant LN	0.79 (0.54,1.16)	0.23	0.91 (0.63,1.31)	0.61
Lung/Pleura	1.06 (0.65,1.73)	0.8	1.09 (0.68,1.73)	0.73
Liver	1.27 (0.85,1.89)	0.25	1.15 (0.78,1.70)	0.47
Peritoneum	1.34 (0.78,2.32)	0.29	1.01 (0.58,1.73)	0.98
Bone	1.67 (1.06,2.63)	**0.028**	1.61 (1.03,2.51)	**0.038**
Brain	0.49 (0.07,3.53)	0.48	1.63 (0.40,6.63)	0.5
SUVmax	0.99 (0.96,1.01)	0.33	1.00 (0.97,1.02)	0.86
SUVmean	0.96 (0.91,1.01)	0.15	0.99 (0.94,1.04)	0.6
SUVpeak	0.98 (0.95,1.01)	0.26	0.99 (0.96,1.02)	0.68
SULmax	0.99 (0.96,1.02)	0.5	1.00 (0.97,1.04)	0.83
SULmean	0.96 (0.89,1.03)	0.23	0.99 (0.92,1.07)	0.86
SULpeak	0.98 (0.94,1.02)	0.4	1.00 (0.96,1.04)	0.99
BMI (kg/m^2^)	0.97 (0.93,1.02)	0.21	0.98 (0.94,1.02)	0.3
SMI (cm^2^/m^2^)	0.97 (0.95,0.99)	**0.0075**	0.97 (0.96,0.99)	**0.011**
Sarcopenia (yes)	1.51 (1.03,2.22)	**0.033**	1.55 (1.07,2.25)	**0.021**
CT features				
NGLDM_Coarseness	0.79 (0.65,0.96)	**0.018**	0.78 (0.64,0.95)	**0.013**
NGLDM_Contrast	0.78 (0.64,0.94)	**0.009**	0.80 (0.67,0.96)	**0.016**
GLZLM_ZP	0.83 (0.69,0.99)	**0.039**		
PET features				
SHAPE_Volume_mL	1.17 (1.00,1.36)	**0.049**	1.22 (1.04, 1.44)	**0.017**
SHAPE_Volume_vx			1.18 (1.00,1.38)	**0.049**
SHAPE_Surface_mm^2^			1.18 (1.01,1.39)	**0.043**
GLZLM_LZE			1.22 (1.02,1.45)	**0.026**
GLZLM_LZLGE	1.21 (1.01,1.46)	**0.044**	1.20 (1.00,1.44)	**0.046**
GLZLM_SZLGE	1.23 (1.01,150)	**0.043**		
40_ SHAPE_Volume_mL			1.20 (1.01,1.42)	**0.038**
70_Kurtosis			1.25 (1.01,1.54)	**0.042**
70_Excess Kurtosis			1.25 (1.01,1.54)	**0.042**

**Table 3:** Multivariable Cox regression analysis for OS and PFS in the overall cohort.

Covariate	OS		PFS	
	HR (95%CI)	*p*-value	HR (95%CI)	*p*-value
Age	1.01 (1.00,1.03)	0.13		
ECOG		**<0.001**		**<0.001**
0–1	reference		reference	
2–3	2.81 (1.65,4.79)		2.65 (1.63,4.30)	
Bone		**0.021**		**0.005**
No	reference		reference	
Yes	1.93 (1.22,3.04)		1.71 (1.09,2.69)	
SMI (cm^2^/m^2^)	0.98 (0.96,1.00)	**0.033**	0.98 (0.96,1.00)	**0.04**
CT features				
NGLDM Coarseness	0.70 (0.53,0.92)	**0.011**		
NGLDM Contrast			0.79 (0.65,0.94)	**0.01**
PET features				
GLZLM SZLGE	1.37 (1.12,1.67)	**0.002**		
SHAPE Volume vx			1.19 (1.01,1.40)	**0.04**
70_ Kurtosis			1.24 (1.00,1.53)	**0.05**

## Data Availability

The data can be shared up on request.
